# Delineating the role of c-FLIP/NEMO interaction in the CD95 network via rational design of molecular probes

**DOI:** 10.1186/s12864-019-5539-y

**Published:** 2019-05-08

**Authors:** Nikita V. Ivanisenko, Jörn H. Buchbinder, Johannes Espe, Max Richter, Miriam Bollmann, Laura K. Hillert, Vladimir A. Ivanisenko, Inna N. Lavrik

**Affiliations:** 1 The Federal Research Center Institute of Cytology and Genetics SB RAS, Novosibirsk, Russia; 2 Translational Inflammation research, Medical Faculty, Otto von Guericke University, Magdeburg, Germany; 3 Novosibirsk State University, Novosibirsk, Russia

**Keywords:** In silico, Homology modeling, Death receptor network, Evolutionary conservation, C-FLIP, V-FLIP, NEMO, NF-κB

## Abstract

**Background:**

Structural homology modeling supported by bioinformatics analysis plays a key role in uncovering new molecular interactions within gene regulatory networks. Here, we have applied this powerful approach to analyze the molecular interactions orchestrating death receptor signaling networks. In particular, we focused on the molecular mechanisms of CD95-mediated NF-κB activation and the role of c-FLIP/NEMO interaction in the induction of this pathway.

**Results:**

To this end, we have created the homology model of the c-FLIP/NEMO complex using the reported structure of the v-FLIP/NEMO complex, and rationally designed peptides targeting this complex. The designed peptides were based on the NEMO structure. Strikingly, the experimental in vitro validation demonstrated that the best inhibitory effects on CD95-mediated NF-κB activation are exhibited by the NEMO-derived peptides with the substitution D242Y of NEMO. Furthermore, we have assumed that the c-FLIP/NEMO complex is recruited to the DED filaments formed upon CD95 activation and validated this assumption in silico. Further insight into the function of c-FLIP/NEMO complex was provided by the analysis of evolutionary conservation of interacting regions which demonstrated that this interaction is common in distinct mammalian species.

**Conclusions:**

Taken together, using a combination of bioinformatics and experimental approaches we obtained new insights into CD95-mediated NF-κB activation, providing manifold possibilities for targeting the death receptor network.

## Background

Continuous growth of the number of available protein crystal structures as well as advances in the crystallization of supramolecular protein complexes make in silico structural modeling techniques a valuable tool in uncovering new molecular mechanisms of the signaling pathways regulation. One of the key signaling networks that attracts major attention in biomedical research is the regulation of the anti-apoptotic and pro-apoptotic pathways in death receptor (DR) signaling. Delineating the molecular mechanisms within this network via advanced structural modeling opens new horizons for getting new insights into its control and pharmacological targeting.

**Fig. 1:**
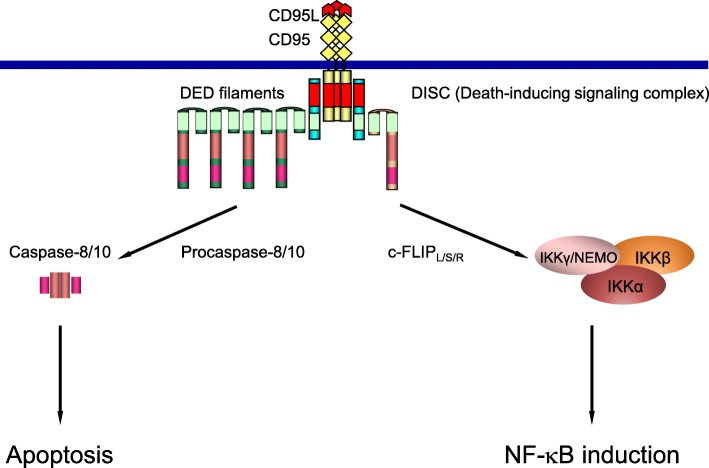
Scheme of CD95 signaling. CD95-mediated induction of apoptotic and anti-apoptotic pathways is shown

CD95/Fas is a member of the DR family, which is a subfamily of the tumor necrosis factor receptor superfamily [1]. Activation of CD95 initiates the extrinsic apoptosis pathway. The CD95-induced apoptotic signal is mediated via the formation of a death-inducing signaling complex (DISC), which comprises CD95, FADD, procaspases-8, − 10 and c-FLIPs (cellular FLICE-like inhibitory proteins) (Fig. 1). The DISC serves as a central platform for procaspase-8 activation, which subsequently initiates an apoptotic cascade [1]. Recently, it has been demonstrated that, at the DISC, procaspase-8 proteins form so-called DED (Death Effector Domain) chains or filaments via interactions of their DED motifs (hereafter termed “filaments”) (Fig. 1). Those serve as a platform for dimerization and subsequent activation of procaspase-8 [2–4].

Stimulation of CD95 not only induces cell death, but can also lead to the activation of NF-κB anti-apoptotic pathway [5–7] (Fig. 1). NF-κB activation is mediated via activation of the IKK complex comprising IKKα/β and the regulatory subunit NEMO (nuclear factor (NF)-κB essential modulator) [8]. NEMO plays a key role in NF-κB signaling control [9]. CD95-mediated NF-κB induction initiates the transcription of anti-apoptotic genes and thereby can block apoptotic cell death (Fig. 1). It was also shown that CD95-mediated NF-κB activation induces the secretion of cytokines that attract phagocytes which clear apoptotic dying cells in vivo [10]. However, the detailed molecular mechanisms of CD95-mediated NF-κB activation are not known yet.

c-FLIP proteins have been reported to play an important role in NF-κB induction and, in particular, in CD95-mediated NF-κB induction [11]. Furthermore, it was shown that the cleavage products p43-FLIP and p22-FLIP, but not non-cleavable c-FLIP_L_ mutants activate the NF-κB pathway [5, 8]. p22-FLIP and p43-FLIP have been reported to interact with the IKK complex [5, 8]. Furthermore, p43-FLIP has been described to be essential for NF-κB induction by CD95 [5]. Additionally it has been reported that upon CD95 stimulation p43-FLIP recruits TRAF2, which in turn links it to NF-κB activation [10]. However, the detailed mechanism of this pathway and the role of p43-FLIP-IKK interaction in NF-κB induction have not been fully deciphered, yet.

**Fig. 2:**
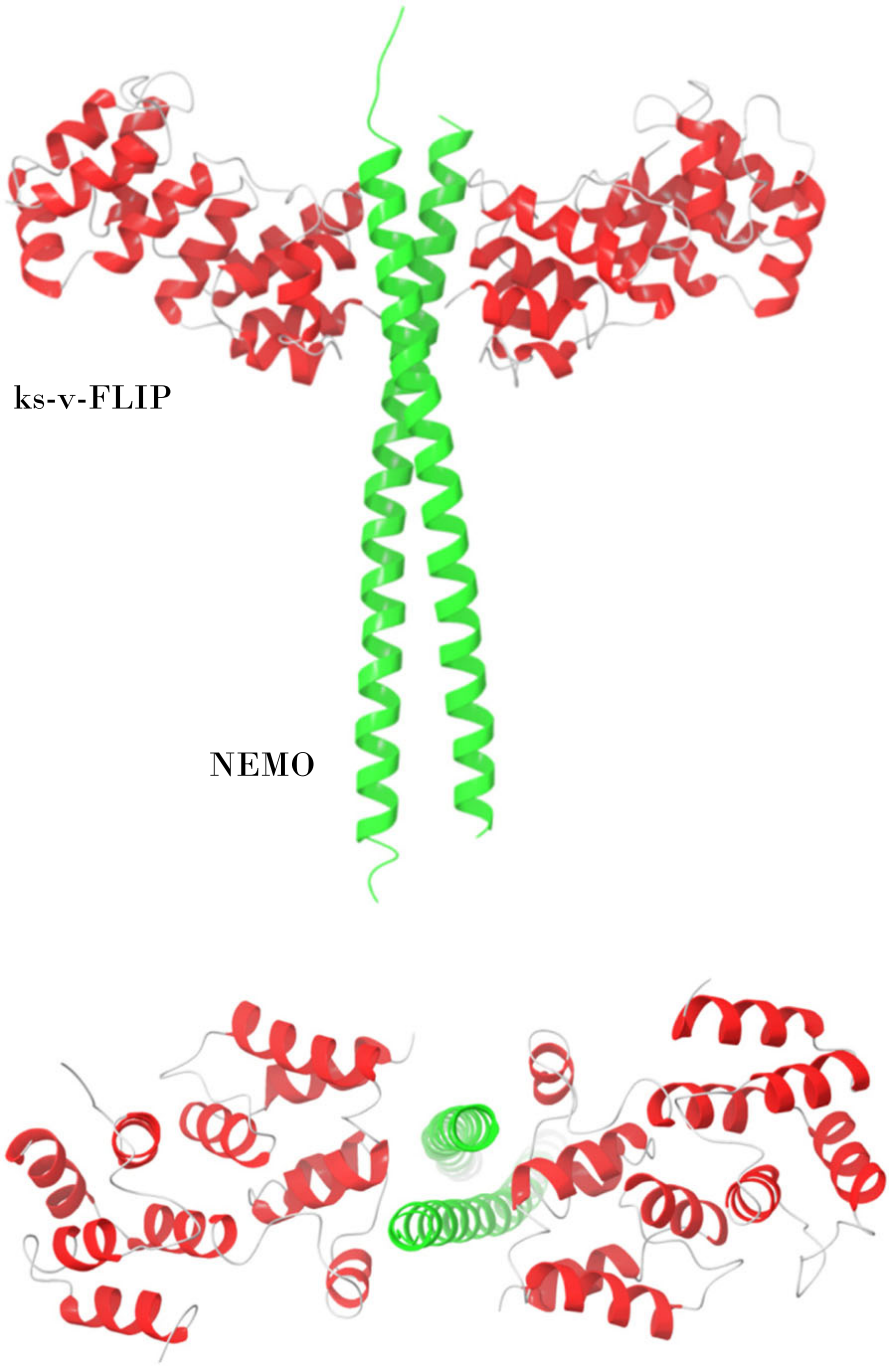
Crystal structure of NEMO and ks-v-FLIP complex (PDB identifier 3CL3). NEMO is shown in green. ks-v-FLIP is presented in red. Two projections of the structure are shown

Importantly, it was shown for viral FLIPs (v-FLIPs) that they directly bind to NEMO thereby activating the NF-κB pathway [12–14]. Furthermore, for ks-v-FLIP (expressed by the Kaposi’s sarcoma herpes virus (KSHV)), a crystal structure of its complex with NEMO was described (Fig. 2). The interaction of ks-v-FLIP with the central region of NEMO (amino acids 150–272) plays a major role in the induction of NF-κB activation [12]. Hence, the question arises whether similar interactions are essential for c-FLIP-mediated NF-κB activation.

To address the question whether the putative interactions between c-FLIP proteins and NEMO play a role in CD95-mediated NF-κB activation, we used structural homology modeling, the design of rational molecular probes combined with experimental validation. In particular, we have designed NEMO-derived peptides based on the structure of the ks-v-FLIP/NEMO complex and addressed their role in CD95-mediated NF-κB activation.

## Results

### Molecular modeling of NEMO and human c-FLIP interaction

**Fig. 3:**
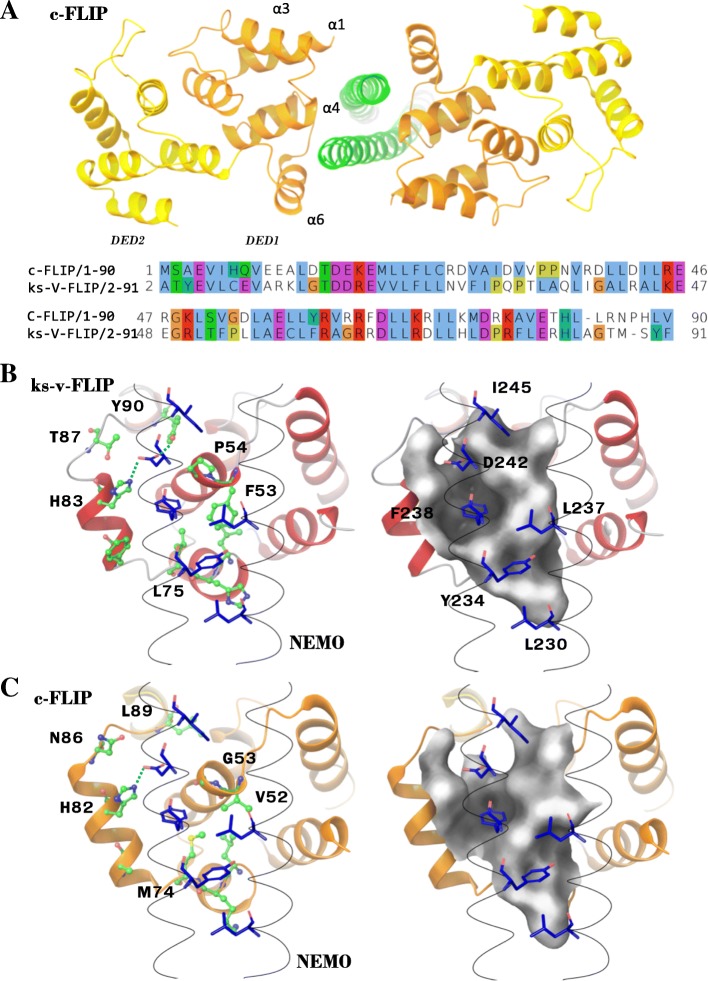
Homology model of NEMO/c-FLIP complex. **a** The homology model of c-FLIP/NEMO complex is shown. NEMO is depicted in green; DED1 and DED2 of c-FLIP are presented in orange and yellow, respectively. The sequence alignment of c-FLIP and ks-v-FLIP DED1 domains is colored according to ClustalX color scheme of Jalview software. **b** ks-v-FLIP/NEMO binding interface. Amino acid residues involved in interaction of ks-v-FLIP (depicted in green color) with NEMO (depicted in blue color) are shown. **c** c-FLIP/NEMO binding interface. Amino acid residues involved in interaction of c-FLIP (depicted in green color) with NEMO (depicted in blue color) are shown. Molecular surface is shown in gray color. For B) and C): c-FLIP and v-FLIP residues are designated at the left side of figure, while NEMO residues at the right side. Hydrogen bonds with NEMO D242 residue are shown with green dashed line

ks-v-FLIP interacts with the NEMO protein through the binding of ks-v-FLIP DED1 with the dimerized 227–248 NEMO region as mentioned above [12] (Fig. 2). Human c-FLIP and viral ks-v-FLIP have a sequence similarity between their DED1 domains of 32.6% (Fig. 3a). To find out whether c-FLIP might also interact with NEMO, we constructed the homology model of a putative c-FLIP/NEMO complex. For this, the available crystal structure of ks-v-FLIP protein in a complex with NEMO was used (PDB identifier 3CL3) (Fig. 3a).

The comparison of structural models of c-FLIP/NEMO and ks-v-FLIP/NEMO shows that both c-FLIP and ks-v-FLIP have a binding pocket which interacts with the F238 and D242 residues of NEMO (Fig. 3b, c). This pocket is formed by amino acid residues H83, L75, V52 and P54 of ks-v-FLIP, while for c-FLIP L75 is replaced by M74 and P54 is substituted by G53 (Fig. 3 b, c). The substitution of P54 to G53 in c-FLIP increases the surface of the putative NEMO interacting site, e.g. increasing the volume of the binding pocket. In both structures a hydrogen bond between the side chain of D242 of NEMO with the conserved H83/H82 residue of the ks-v-FLIP/c-FLIP proteins is observed. In contrast, the second hydrogen bond of ks-v-FLIP Y90 with the side chain of D242 is no longer detected in the c-FLIP/NEMO complex (Fig. 3c). One can expect that this substitution leads to decreased NEMO binding affinity of c-FLIP in comparison with v-FLIP. The overall structural comparison of binding interfaces allowed formulating the hypothesis that both, c-FLIP and v-FLIP, have a NEMO binding site (Fig. 3b, c).

**Fig. 4:**
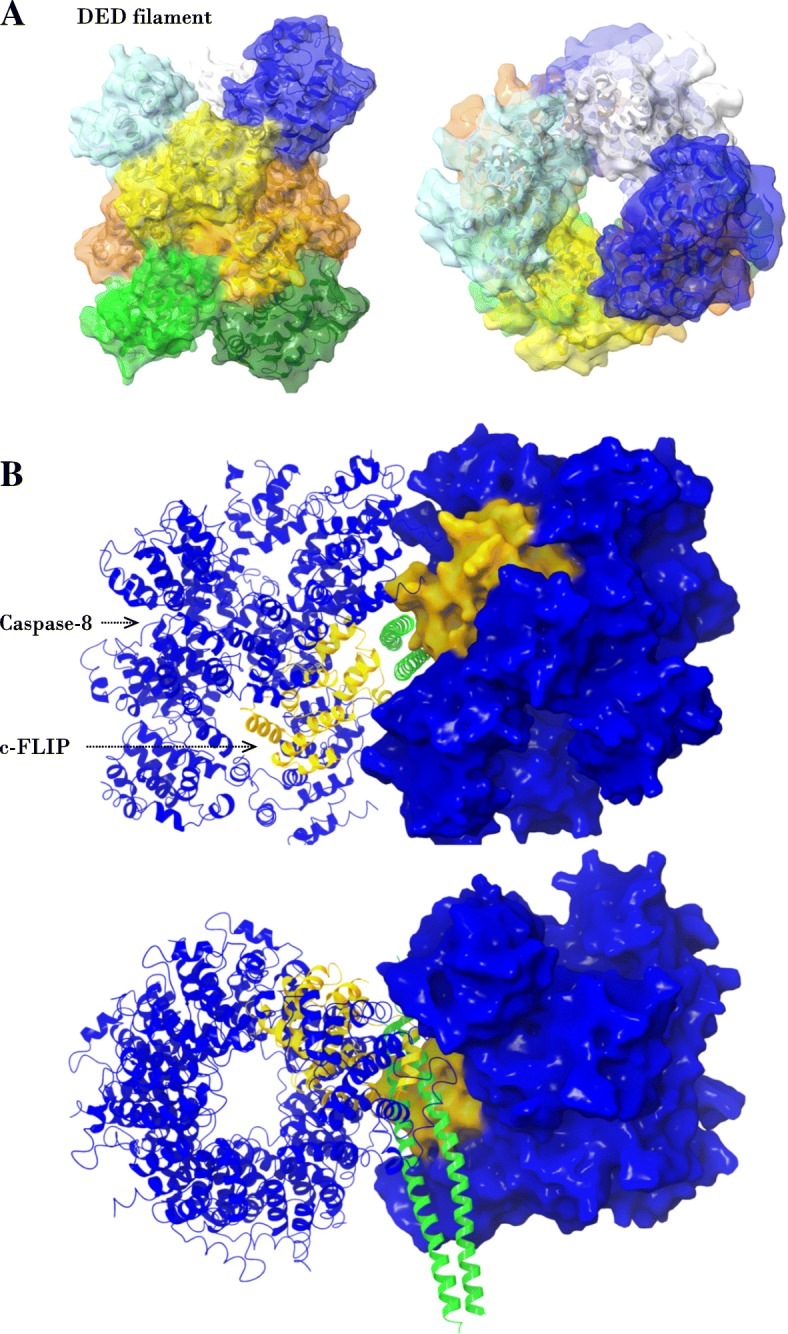
NEMO binds simultaneously to two caspase-8/c-FLIP DED filaments. **a** caspase-8 DED filament (PDB identifier 5 l08) is shown in two projections. **b** The homology-based of NEMO interaction with two caspase-8 (blue color) and c-FLIP (yellow color) DED filaments. Two projections are shown

CD95 stimulation leads to the formation of DED filaments at the DISC, to which c-FLIP proteins are subsequently recruited. Hence, upon CD95 stimulation it is natural to expect the recruitment of NEMO to the filaments via its putative interactions with c-FLIP. To test this hypothesis, we analyzed if NEMO binding to c-FLIP may affect DED filament formation and c-FLIP interactions in the DED filament. We also tested this assumption considering that NEMO remains in the dimeric configuration. For this we used a tertiary structure obtained from the available EM structure of the caspase-8 DED filament (PDB identified 5 L08, [4]). According to the EM structure each DED domain can interact with up to six neighboring DED domains (Fig. 4a). Surprisingly, a structural superimposition of the c-FLIP/NEMO dimer complex shows that NEMO binding does not affect any type of DED/DED interactions (Fig. 4b). Moreover, the structural alignment shows that the NEMO dimer can simultaneously bind to two DED filaments containing c-FLIP, while the spatial orientations between two filaments is adapted in such a way that no sterically forbidden contacts arise (Fig. 4b). It is suggested that a simultaneous binding of two DED filaments would lead to an additional stabilization of the c-FLIP/NEMO complex by interactions between polar residues of NEMO and DED filaments (Fig. 4b). Taken together, this analysis shows that upon CD95 stimulation NEMO can potentially bind to c-FLIP in the DED filament and that this interaction can be stabilized by its dimeric structure.

### Rational design of NEMO-based c-FLIP binding peptides

To investigate a functional role and validate the binding of c-FLIP and NEMO we proceeded with a rational design of molecular probes. The putative binding site at NEMO represents an alpha-helical domain. This provided an important basis for the design of NEMO-based peptides. Indeed, the construction of the probes based on short alpha-helical peptides has proven to be an effective strategy to identify inhibitors of protein-protein interactions. In particular, recently it has been utilized by [15] to inhibit the interaction of ks-v-FLIP with NEMO.

**Fig. 5:**
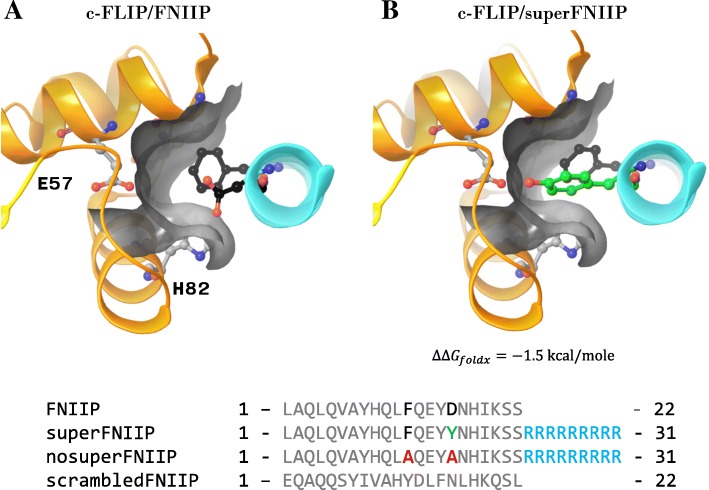
Design of NEMO-based c-FLIP binding and control peptides. Predicted structures of c-FLIP/FNIIP (**a**) and c-FLIP/superFNIIP (**b**) complexes. The peptide sequences are shown on the bottom of figure

We started the design of c-FLIP targeting peptides from the sequence of the binding interface of the c-FLIP/NEMO complex, (Nemo sequence: 227–248 LAQLQVAYHQLFQEYDNHIKSS) (Fig. 5). Interestingly*,* estimations of binding energies using FoldX showed that this peptide fragment has an increased binding affinity to ks-v-FLIP (− 14.6 kcal/mole) in comparison with c-FLIP (− 12.5 kcal/mole). One of the peculiar differences in NEMO binding regions between c-FLIP and ks-v-FLIP is the lack of a tyrosine residue (ks-v-FLIP Y90) in the corresponding c-FLIP fragment. Y90 is able to form a hydrogen bond with D242 of NEMO, which potentially stabilizes the complex. Additionally, an increased surface of the binding interface in the vicinity of D242 binding site is observed due to the presence of glycine in c-FLIP (G53) instead of proline in ks-v-FLIP (P54). For these reasons we have decided to optimize the NEMO-derived peptide sequence (Fig. 5).

To identify residues that are able to increase the binding affinity of the NEMO-peptide to c-FLIP we used the FoldX software [16]. We scanned for all amino acids which can increase the predicted binding affinity of proteins and at the same time did not decrease the stability of the unbound peptide. In this way, the substitution D242Y was identified. According to the molecular model it leads to the formation of an additional hydrogen bond with carboxylic acid of E57 of c-FLIP and can efficiently occupy the space between the H82 and G53 amino acid residues of c-FLIP. The predicted difference in stability of the peptide interaction as calculated by FoldX was − 1.2 kcal/mole. Hence, this generated peptide was used for the subsequent analysis and termed superFNIIP (Fig. 5).

To further validate the interaction of NEMO peptide and c-FLIP we designed two control peptides. First, we used a scrambled sequence of NEMO (227–248) to generate a peptide with similar physico-chemical properties termed scrFNIIP. This peptide is expected to have no possibility to bind to c-FLIP. Second, a control peptide was designed by introduction of two mutations, F238A and D242A, into the NEMO (227–248) sequence. Based on the molecular model this peptide should have a significantly reduced affinity to c-FLIP due to replacement of the F238 and D242 residues involved in recognition of the putative c-FLIP pocket (nosuperFNIIP) (Fig. 5).

### NEMO-derived peptides block CD95-mediated NF-κB induction

**Fig. 6:**
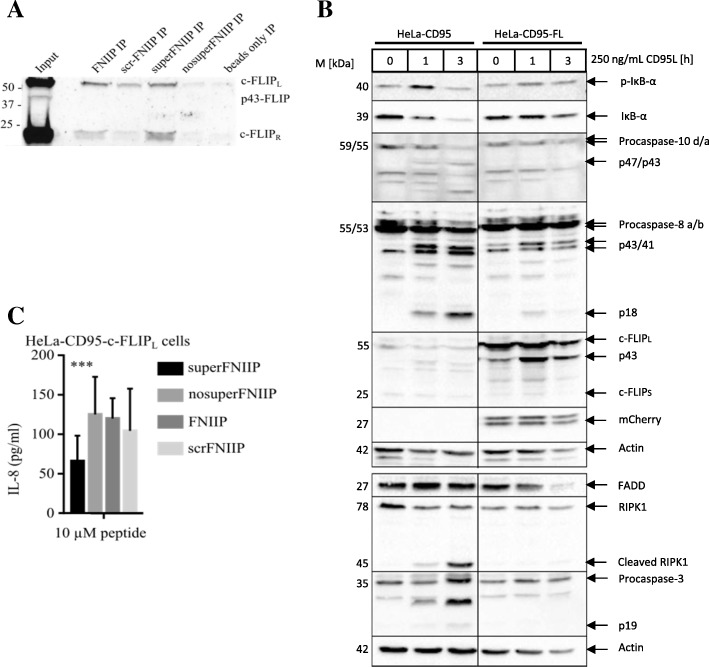
Experimental validation of the peptides. **a** Peptides were covalently bound to beads and incubated with lysates from HeLa-CD95-FRL cells. Binding of c-FLIP was analyzed with immunoblotting for c-FLIP. **b** Apoptotic/non-apoptotic signaling in HeLa-CD95-FL cells upon CD95L stimulation for one and three hours (h) was analyzed via immunoblotting. Immunoblotting has been performed using the indicated antibodies. As the hallmarks of apoptosis induction the processing of procaspase-8a/b, procaspase-10d/a and procaspase-3 to their cleavage products has been analyzed. As the hallmark of NF-κB activation, the phosphorylation and degradation of IκBα were monitored. Actin was used as a loading control. One representative experiment out of three independent experiments is shown. **c** Analysis of the peptide effects on CD95-mediated NF-κB activation via IL8 ELISA analysis

As a first step of experimental validation, it was investigated whether the designed peptides bind to their targets, e.g. the c-FLIP proteins. This was carried out via a pull-down assay in c-FLIP_L/R_ overexpressing cells, which were described by us before [5]. The superFNIIP peptide was able to bind to c-FLIP_L_ and c-FLIP_R_ while nosuperFNIIP showed no binding to c-FLIP (Fig. 6a). FNIIP has also demonstrated the binding to c-FLIP, albeit less efficiently compared to the superFNIIP peptide (Fig. 6a). Both isoforms c-FLIP_L_ and c-FLIP_R_ have DED1 and DED2 in their structure, while c-FLIP_L_ in addition possesses the C-terminal domain. Hence, this pull-down assay demonstrates that the NEMO peptides interact with both c-FLIP isoforms, underlining the very likely involvement of the N-terminal DED-containing part in this interaction. The latter is in full accordance with the peptide design. Taken together, these results confirmed that the designed peptides specifically interact with c-FLIP proteins.

We suggested that the addition of the NEMO-derived peptide would inhibit the c-FLIP/NEMO interaction. This subsequently would result in a decrease of CD95-mediated NF-κB activation. This suggestion was evaluated by analyzing IL-8 secretion after 24 h of stimulation with 250 ng/ml CD95L in HeLa-CD95-FL cells (HeLa-CD95 cells overexpressing c-FLIP_L_). This cell line has been generated by us recently and is characterized by a stable overexpression of c-FLIP_L_ as well as apoptotic and non-apoptotic CD95 signaling. The latter is illustrated by the analysis of the effects of CD95 stimulation via immunoblotting (Fig. 6b). IL8 is an NF-κB target gene and its expression has been reported to be one of the key assays for testing CD95-mediated NF-κB activation [10]. The peptides superFNIIP, nosuperFNIIP, FNIIP and scrFNIIP were added to the cells 30 min before stimulation with CD95L. Importantly, in these experiments the pan-caspase inhibitor zVAD-fmk was used to prevent CD95-induced cell death. The use of the peptide superFNIIP showed decreased IL-8 secretion versus the control peptide nosuperFNIIP (Fig. 6c). Thus, we could show that the designed peptide decreased CD95-mediated NF-κB activation, which subsequently supports our hypothesis. Furthermore, the substitution of D242 to Y in the NEMO-derived peptide seems to have a much stronger effect on CD95-mediated NF-κB activation, further supporting the hypothesis of differences in the stability of c-FLIP/NEMO vs. v-FLIP/NEMO complexes.

### The c-FLIP/NEMO interaction is conserved in mammalian organisms

The c-FLIP protein is known to be a multifunctional regulator of programmed cell death. From one side, it is a well-known inhibitor of caspase-8 activation and from another side it might be essential for NF-κB activation. After proposing that c-FLIP/NEMO interaction plays a role in the regulation of CD95-mediated NF-κB activation at the DED filament, next we aimed to address whether this role of c-FLIP has appeared earlier in evolution than its role as an inhibitor of caspase-8 activation.

**Fig. 7:**
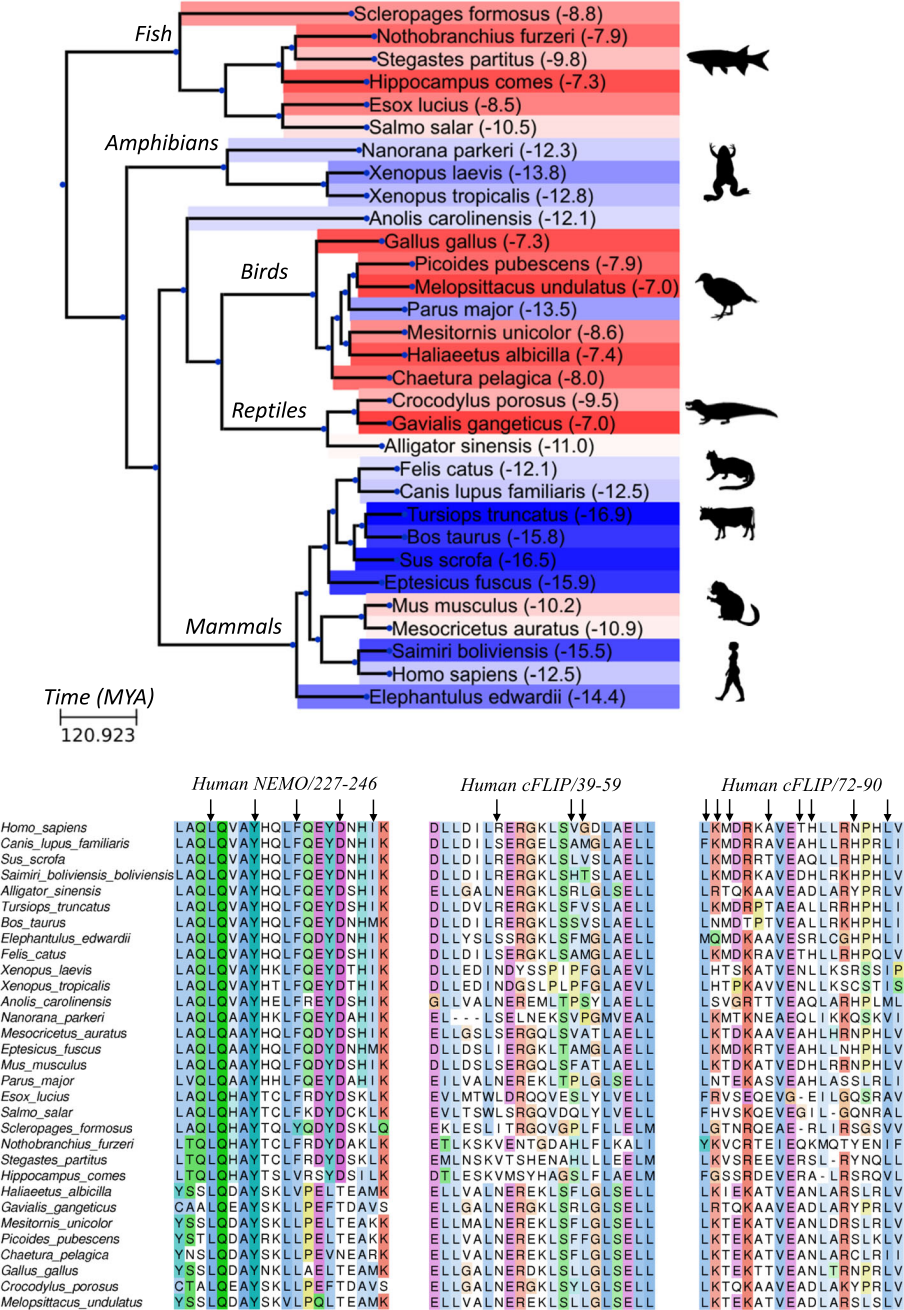
Analysis of evolutionary conservation of c-FLIP/NEMO binding sites. Phylogeny of the species and time points of the lineage diversification calculated using TimeTree database [23] are shown on the top of the figure. Background of the branches is colored based on predicted binding energy of c-FLIP and NEMO using FoldX. Reference organism names and predicted binding energies are indicated. Blue color gradient indicates an increase of stability of a complex in comparison with human complex; Red gradient indicates a decrease in stability. Silhouette images were obtained from PhyloPic (http://phylopic.org). The sequence alignments of NEMO and c-FLIP binding site regions are shown on the bottom of the figure. Arrows indicate amino acid residues located on the binding site interface of c-FLIP and NEMO

To address this question we analyzed whether c-FLIP/NEMO interaction is evolutionary conserved in higher vertebrates. Genomes of higher vertebrates were found to encode proteins homologous to c-FLIP and NEMO. Moreover, the sequence of the human NEMO region (227–248) was highly conserved among multiple organisms, underlining its important role in regulation of the anti-apoptotic pathway (Fig. 7). On the contrary, the c-FLIP regions involved in NEMO binding were found to be more variable. This could indicate that the interaction of c-FLIP and NEMO is not critical for regulation of the NF-κB pathway in those organisms where both c-FLIP and NEMO are present.

To further address this question and address the relevance of c-FLIP/NEMO interaction we estimated a binding energy of identified homologous c-FLIP/NEMO complexes using the FoldX software. Most of the mammalian species had a binding energy similar to that in *Homo sapiens* (Fig. 7). The most stable binding was observed in *Cetartiodactyla*, including *Bos taurus* (cow) and *Tursiops truncates* (dolphin*)*, while *Rodentia,* including *Mus musculus* (mouse) and *Mesocricetus auratus* (golden hamster) had a reduced binding energy. A surprisingly similar binding energy of c-FLIP and NEMO was predicted in amphibian species: *Xenopus laevis, Xenopus tropicalis* and *Nanorana perkeri,* while for most of the birds and fish species the binding energy of the c-FLIP/NEMO interaction was significantly lower (Fig. 7). Remarkably, for most bird species the NEMO homologous protein had a higher similarity to human Optineurin rather than to human NEMO. Human Optineurin has a high similarity to NEMO, however it does not interact with IKKα/IKKβ [17].

Predicted low c-FLIP/NEMO binding energies among fish species together with estimations of the time points of lineage diversification allow to conclude that the regulation of the NF-κB pathway by c-FLIP could have appeared later in the course of evolution than its function in the apoptotic signaling. Moreover, we can assume that the c-FLIP/NEMO regulation pathway played a key role in the development of some amphibians and mammals, while birds and reptiles utilized a different cell death regulation strategy.

## Discussion

In this study, we have investigated the role of the c-FLIP/NEMO interaction in the CD95 network using the state of the art technology of homology modeling, rational peptide design, and the analysis of evolutionary conservation.

The construction of the homology model allowed to identify key interactions in the c-FLIP/NEMO complex and enabled the rational design of molecular probes. Importantly, we found that the variation of only one amino acid in the sequence of the NEMO–derived peptide, which corresponds to D242 in NEMO sequence, can significantly improve the binding of NEMO-derived peptide to c-FLIP. Indeed, we have started our study with designing peptides without these substitutions and we could not provide any experimental evidence that these initially constructed peptides play any role in CD95-mediated NF-κB activation (data not shown). These results provide indirect evidence for the importance of the identified molecular interactions. Furthermore, these findings suggest that ks-v-FLIP/NEMO interaction is more stable compared to c-FLIP/NEMO, which accordingly might result in the less efficient activation of NF-κB via c-FLIP proteins compared to ks-v-FLIPs.

The role of the c-FLIP/NEMO interaction in CD95-mediated NF-κB activation has been controversially discussed. Some studies suggest that c-FLIP/NEMO interaction is not essential for NF-κB activation [14]. However, our computational analysis and modeling strongly suggest that this interaction takes place and might play a role particular in the CD95-mediated NF-κB activation. c-FLIP proteins are essential components of the DED filaments and therefore they might also recruit NEMO to DED filaments followed by the recruitment of the IKK complex and NF-κB activation. The in silico analysis performed by us shows that there are additional possibilities for the stabilization of c-FLIP/NEMO complex in the DED filament structure that would further support the role of this interaction in particular, in CD95-mediated NF-κB pathway. This might be a major difference to ks-v-FLIP/NEMO complex. Furthermore, it was shown before that ks-v-FLIP block DED filament formation [18], which further supports different roles of ks-v-FLIP/NEMO vs. c-FLIP/NEMO interactions. Interestingly, there are a number of reports suggesting the formation of DR-induced complexes including procaspase-8, IKK and the components of the NF-κB pathway that drive NF-κB induction [19]. Our study suggests that c-FLIP might serve as a key link between recruitment of these complexes into DED filaments and induction of the NF-κB pathway.

Importantly, we have shown in our previous studies using quantitative proteomics analysis that c-FLIP proteins are characterized by a low abundance in the DED filaments [18, 20]. The model of CD95-mediated NF-κB activation proposed by us, which is based on c-FLIP/NEMO interaction would support the low strength of CD95-induced NF-κB activation. This has been reported by us and others and further highlights the difference between CD95- and other DR- mediated NF-κB activation such as TNFα-mediated NF-κB induction.

It has to be noted that upon addition of NEMO-derived peptide we did not observe a full blockage of CD95-mediated IL8 production in our experiments (Fig. 6c) nor did we observe any strong effects on CD95-mediated p65 translocation to the nucleus and other mediators (data not shown). These observations might be resulting from a low stability of the peptides in the cell. Crosslinking of these peptides might significantly improve their pharmacological properties and will be investigated in the future experiments. Another explanation for the moderate effect on the pathway might be the stoichiometry of the NEMO interactions. Based on the structural model, self-dimerization of NEMO-derived peptides can be essential to form a stable complex with DED filaments. The latter would require higher cellular concentrations of NEMO-derived peptides to achieve a functional effect, which can be limited by the level of penetration of peptides into cell.

An interesting twist to the study was given by the analysis of the evolutionary conservation of the site of c-FLIP/NEMO interaction. Based on the estimation of the stability of the c-FLIP/NEMO complex we can assume that this interaction appeared later in evolution than interactions of c-FLIP leading to apoptosis inhibition. Interestingly, we can suggest that this interaction is of a major importance in most of the mammalian organisms, while birds and reptiles apparently do not use this interaction in the regulation of cell death (Fig. 7).

The understanding of the crosstalk between DR-induced apoptotic and anti-apoptotic pathways is essential for the success of anti-cancer therapies based on the activation of the DR pathway. Indeed, DR stimulation might also induce a strong anti-apoptotic response, which naturally might prevent apoptosis by upregulation of the anti-apoptotic genes and, therefore, counteract the effect of anti-cancer therapies. Thus, construction of rationally designed probes based on the selective inhibition of the DR-NF-κB pathway as suggested in this study plays a very important role in the future development of specific anticancer therapies.

## Conclusions

Taken together, using the latest bioinformatical approaches supported by the experimental analysis, we have uncovered the role of c-FLIP/NEMO interaction in CD95 network. In particular, we have demonstrated that this interaction might play a major role in particular in CD95-mediated NF-κB induction. Furthermore, by using evolutionary conservation analysis we have demonstrated that this interaction could have appeared relatively late in evolution and involved in cell death regulation of mammalian species. These findings provide new opportunities for the design of new specific anti-cancer therapies based on the inhibition of death receptor-mediated anti-apoptosis pathways.

## Methods

### Homology modeling

Homology modeling of DED domains of human c-FLIP was conducted using Modeller 9v12 [21]. The model with the best DOPE score among 1000 generated models was selected. The crystal structure of ks-v-FLIP in complex with NEMO was used as a template for homology modeling (PDB identifier 3CL3; [12]). Multiple sequence alignments of DED domains were obtained using the Clustal Omega program and included c-FLIP, ks-v-FLIP and procaspase-8 sequences [22]. The structure of the c-FLIP/NEMO complex was obtained by the structural superimposition of the homology model of c-FLIP on the ks-v-FLIP/NEMO crystal structure. The molecular model of c-FLIP/procaspase-8 DED filament was obtained by structural superimposition and replacement of one of the procaspase-8 subunits. Structural superimposition was conducted using the Schrödinger suite software (maestro, version 2017–2). The binding interface of the homology model of c-FLIP/NEMO was optimized using the FoldX RepairPDB module [16].

### Binding energy estimation

Amino acid substitutions were introduced using the FoldX BuildModule module [16]. Analysis of protein binding energies was carried out using the FoldX Stability module and was calculated as the difference between the stability of bound and unbound components [16].

### Analysis of evolutionary conservation of c-FLIP/NEMO binding sites

Sequences of NEMO and c-FLIP for different organisms were obtained using BLAST search against the NCBI NR (non-redundant set) database of human NEMO (150–350) and c-FLIP (1–170) regions. For each organism c-FLIP sequences with the lowest E-value were selected. NEMO sequences with the lowest E-value and full sequence coverage of NEMO (227–247) regions were selected. Reference organisms with both identified homologous c-FLIP and NEMO (227–247) sequences were considered for analysis. Multiple sequence alignment was created using the Clustal Omega program. The phylogeny of the species and time points of lineage diversification were calculated using the TimeTree database [23]. The Ete3 python script was used for visualization of the taxonomic tree [24]. For each analyzed organism amino acid substitutions located on the binding interface of the c-FLIP and NEMO were introduced using FoldX in the reference structure of human c-FLIP/NEMO complex according to sequence alignment. The binding energies of c-FLIP and NEMO in obtained complexes were estimated using the FoldX software. GenBank identifiers of the analyzed sequences are shown in Table 1.

**Table 1:** GenBank identifiers of the analyzed sequences

Organism	NEMO, GenBank ID	c-FLIP, GenBank ID
*Salmo salar*	XP_014003062.1	XP_014042947.1
*Mesitornis unicolor*	XP_010184587.1	XP_010188337.1
*Haliaeetus albicilla*	XP_009921898.1	KFP93394.1
*Esox lucius*	XP_010903123.1	XP_019897090.1
*Stegastes partitus*	XP_008302258.1	XP_008285877.1
*Nanorana parkeri*	XP_018417322.1	XP_018425650.1
*Sus scrofa*	XP_020935030.1	AAS22336.1
*Nothobranchius furzeri*	XP_015824373.1	XP_015822170.1
*Anolis carolinensis*	XP_008102141.1	XP_008118737.1
*Xenopus laevis*	NP_001089998.1	OCT63813.1
*Parus major*	XP_015471624.1	XP_015489701.1
*Xenopus tropicalis*	NP_001120221.1	XP_012826880.1
*Alligator sinensis*	XP_006029398.2	XP_006022153.1
*Saimiri boliviensis boliviensis*	XP_010330107.1	XP_003925710.1
*Melopsittacus undulatus*	XP_005143390.1	XP_005145602.2
*Scleropages formosus*	XP_018580828.1	XP_018585030.1
*Homo sapiens*	NP_003630.1	AAC16441.1
*Picoides pubescens*	XP_009898568.1	XP_009894784.1
*Canis lupus familiaris*	XP_013967102.1	XP_022270683.1
*Bos taurus*	XP_010819849.1	XP_024855306.1
*Mus musculus*	NP_001129539.1	NP_033935.2
*Gallus gallus*	NP_989567.1	XP_015144910.1
*Tursiops truncatus*	XP_019789232.1	XP_019794717.1
*Eptesicus fuscus*	XP_008157513.1	XP_008144686.1
*Felis catus*	XP_019680062.1	XP_023115368.1
*Chaetura pelagica*	KFU92351.1	XP_010005644.1
*Mesocricetus auratus*	XP_012981324.1	XP_005070664.2
*Crocodylus porosus*	XP_019399239.1	XP_019409416.1
*Gavialis gangeticus*	XP_019365101.1	XP_019378755.1
*Elephantulus edwardii*	XP_006899976.1	XP_006889143.1
*Hippocampus comes*	XP_019746968.1	XP_019737865.1

### Peptides

Peptides were synthesized by LifeTein LLC (Summerset, NJ, USA) and had at least 95% purity. The N-terminus was acetylated and the C-terminus was amidated for extended stability. LifeTein has performed quality control via mass spectrometry and liquid chromatography analysis. The sequences of the peptides are given in Fig. 5. As a cell-penetrating sequence R9 has been used.

### Cell lines

Human cervical cancer HeLa-CD95 [5] (CD95-overexpressing cells), HeLa-CD95-FRL (CD95/c-FLIP_L_/c-FLIP_R_ -overexpressing cells) [25] and HeLa-CD95-FL cells (CD95/c-FLIP_L_-overexpressing cells) were maintained in DMEM/Ham’s F12 media (Merck Millipore, Germany), supplemented with 10% heat-inactivated fetal calf serum, 1% Penicillin-Streptomycin and 10 ng/ml Puromycin in 5% CO_2_. HeLa-CD95 cells were stably transduced with the Lenti-X Packaging Single Shot (VSV-G) including the pLVX-IRES-mCherry-FLIP_L_ vector. Viral transduction was performed according to the manufacturer’s instructions (Clontech, Germany). Afterwards cells were isolated and sorted according to mCherry fluorescence using FACSAria III (Becton Dickinson).

### Antibodies and reagents

The following antibodies were used for Western Blot analysis: polyclonal anti-caspase-3 antibody (#9662) and monoclonal anti-RIPK1 XP antibody (#3493) from Cell Signaling; polyclonal anti-actin antibody (A2103; Sigma-Aldrich, Germany), polyclonal anti-mCherry antibody (ab183628; Abcam), monoclonal anti-caspase-10 antibody (M059–3; MBL International), monoclonal anti-FADD antibody (clone 1C4), monoclonal anti-caspase-8 antibody (clone C15) and monoclonal c-FLIP antibody (clone NF6). Horseradish peroxidase-conjugated goat anti-mouse IgG1,-2a,-2b, goat anti-rabbit and rabbit anti-goat were from Santa Cruz (California, USA). All chemicals were of analytical grade and purchased from Merck (Darmstadt, Germany) or Sigma-Aldrich (Germany). The C15, NF6 and 1C4 antibodies were kindly provided by Prof. P. H. Krammer (DKFZ, Heidelberg). Recombinant LZ-CD95L was produced as described [25].

### Analysis of total cell lysates by immunoblotting

Immunoblotting of total cellular lysates was performed in accordance to our previous reports [26].

### Immunoprecipitation

Peptides were immobilized on beads using a kit from Pierce according to the manufacturer’s instructions. The subsequent immunoprecipitation (IP) from 5 × 10^6^ HeLa-CD95-FRL cells [25] cells was performed as described before [27]. In addition, IPs were washed four times with PBS, followed by immunoblotting.

### Il-8 ELISA

For ELISA analysis, HeLa-CD95-FL cells were seeded into 96 well plates. On the following day, media were removed, 75 μl fresh medium was added and cells were pre-incubated with 10 μM of the peptides and 50 μM caspase inhibitor zVAD-fmk for 30 min. Afterwards, cells were stimulated with CD95L in a total volume of 150 μl. After 24 h of stimulation, IL-8 levels in the supernatant were measured with an IL-8 ELISA kit (BioLegend, San Diego, USA) according to the manufacturer’s instructions.

## Abbreviations

c-FLIPs: cellular FLICE-like inhibitory proteins
DED: Death Effector Domain
DISC: death-inducing signaling complex
DR: death receptor
KS: Kaposi’s sarcoma
NEMO: nuclear factor (NF)-κB essential modulator
v-FLIPs: viral FLIPs
